# Strategy for the treatment and follow-up of sinonasal solitary extramedullary plasmacytoma: a case series

**DOI:** 10.1186/s13256-017-1382-4

**Published:** 2017-08-10

**Authors:** Elena Cantone, Antonella Miriam Di Lullo, Luana Marano, Elia Guadagno, Gelsomina Mansueto, Pasquale Capriglione, Lucio Catalano, Maurizio Iengo

**Affiliations:** 1 Department of Neuroscience, Reproductive and Odontostomatologic Science, ENT Section, “Federico II” University of Naples-Italy, S. Pansini, 5, 80131 Naples, Italy; 2 Department of Hematology, “Federico II” University of Naples-Italy, S. Pansini, 5, 80131 Naples, Italy; 3 Department of Advanced Biomedical Sciences, Pathology Section, “Federico II” University of Naples-Italy, S. Pansini, 5, 80131 Naples, Italy; 4 Università degli Studi di Napoli “Federico II”, Via Pansini, 5, Naples, Italy

**Keywords:** Plasmacytoma, Sinus, Radiotherapy, Plasma cell, Epistaxis

## Abstract

**Background:**

Extramedullary plasmacytoma is a rare neoplasm characterized by monoclonal proliferation of plasma cells outside bone marrow. It accounts for 4% of all non-epithelial sinonasal tumors. According to the literature, radiotherapy is the standard therapy for extramedullary plasmacytoma. However, the conversion rate of extramedullary plasmacytoma to multiple myeloma is reported to be between 11 and 33% over 10 years. The highest risk of conversion is reported during the first 2 years after diagnosis, but conversion has been noted up to 15 years after diagnosis. Once conversion to multiple myeloma is complete, less than 10% of patients will survive 10 years.

**Case presentation:**

We present three cases of sinonasal extramedullary plasmacytoma who underwent radiotherapy: a 61-year-old white man, a 60-year-old white man, and a 37-year-old white woman. We found long-term survival with stable disease in all three cases.

**Conclusions:**

The management of solitary extramedullary plasmacytomas of the sinonasal tract is not well established yet. However, the possibility of recurrence and progression to multiple myeloma requires a thorough follow-up protocol. Due to the absence of a standardized protocol for these tumors, we tried to design a tailored long-term follow-up scheme.

## Background

Plasmacytoma is a malignant neoplasm of monoclonal B cells first described by Schridde in 1905 [1]. It consists of three distinct entities according to the International Myeloma Working Group, 2003: solitary plasmacytoma of bone (SPB), extramedullary plasmacytoma (EMP), and multiple primary or recurrent plasmacytomas (Table 1) [2, 3].

**Table 1:** International Myeloma Working Group diagnostic criteria of solitary plasmacytoma of bone, extramedullary plasmacytoma, and multiple solitary plasmacytomas (primary or recurrent) [3]

Diagnosis	Criteria
Solitary plasmacytoma of bone (SPB)	No M-protein in serum and/or urine* Single area of bone destruction due to clonal plasma cells Bone marrow not consistent with multiple myeloma (plasma cells <5%) Normal skeletal survey (and magnetic resonance imaging of spine and pelvis if done) No related organ or tissue impairment (no end organ damage other than solitary bone lesion)*
Extramedullary plasmacytoma (EMP)	No M-protein in serum and/or urine* Extramedullary tumor of clonal plasma cells Normal bone marrow Normal skeletal survey No related organ or tissue impairment (end organ damage including bone lesions)*
Multiple solitary plasmacytomas (primary or recurrent)	No M-protein in serum and/or urine* More than one localized area of bone destruction or extramedullary tumor of clonal plasma cells which may be recurrent Normal bone marrow Normal skeletal survey and magnetic resonance imaging of spine and pelvis if done No related organ or tissue impairment (no end organ damage other than the localized bone lesions)

*A small M-component may sometimes be present

Modified by International Myeloma Working Group. “Criteria for the classification of monoclonal gammopathies, multiple myeloma and related disorders: a report of the International Myeloma Working Group.” *Br J Haematol* 2003;121:749–57 [3]

EMP accounts for less than 4% of all plasma cell tumors and represents 1% of all head and neck tumors [4–6]. More than 80% of plasma cell tumors are localized in the upper aero-digestive tract with approximately 80% within the sinonasal cavity [4–6]. In general, the median age at diagnosis of EMP is 55 to 60 years with a male/female ratio of 3:1 [7]. Only a few cases of EMP (15 to 20%) progress to multiple myeloma (MM) [4]; however, despite recent advances in laboratory, imaging, and clinical evaluation, it is still impossible to identify which cases of EMP progress to MM [5].

Nasal obstruction, usually unilateral, is the most common presenting symptom of sinonasal EMP; however, it is observed in only 29.8% of cases [8]. Other commonly presenting symptoms are epistaxis, facial swelling, pain, and rhinorrhea [5]. The presence of cervical lymph nodes involvement at diagnosis ranges from 5 to 20% of cases [5].

A biopsy of the tumor is required to confirm the diagnosis of EMP [7, 9]. It is based on the morphologic and immunophenotypic findings of localized monoclonal plasma cells in the absence of plasma cells proliferation in other sites, especially in the bone marrow, and in the absence of malignant lymphoma [5]. CD138 is the most useful plasma cell marker [7].

Although different therapeutic approaches have been proposed in the literature, due to its high radiosensitivity, radiotherapy represents the treatment of choice for EMP [1, 5]. However, complete surgical excision is suggested only in small localized lesions, while the role of chemotherapy still remains unclear [7].

In this article we reported a series of three sinonasal EMPs (two men, one woman; mean age 53, range 37 to 61) aiming to clarify some diagnostic and therapeutic aspects.

## Case presentation

### Case 1

A 61-year-old white man complained of diffuse right maxillary sinus pain, ipsilateral epistaxis, and rhinorrhea for 4 months. A complete basal serum work-up was normal, including serum and urine protein electrophoresis. A nasal endoscopy showed a bloody tumor mass occupying his right nasal cavity; at clinical examination no palpable laterocervical lymph nodes were found.

A computed tomography (CT) scan revealed the presence of soft tissue involving his right maxillary sinus extending into the ipsilateral nasal cavity with size greater than 4 cm. Signs of bone erosion were observed at the level of alveolar superior bone, with osteolytic area of 2.5 cm extended to his nasal septum.

Magnetic resonance imaging (MRI) with contrast medium showed hyperintense signal in T1 and fluid-attenuated inversion recovery (FLAIR) sequences and hypointense signal in T2 in his right maxillary sinus and nasal fossa.

A nasal biopsy performed under local anesthesia allowed the diagnosis of EMP. An immunohistochemical study showed diffuse positivity for CD138 with immunoglobulin (Ig) kappa light chain restriction. An iliac crest needle biopsy did not demonstrate bone marrow involvement; total body sestamibi scintigraphy showed normal tracer uptake in bones and soft tissues.

He underwent radiotherapy with a total dose of 60 Gy, by linear accelerator, with complete disappearance of disease, but after 5 years he presented with a local recurrence confirmed by a biopsy of the lesion. He was then treated with chemotherapy based on thalidomide and dexamethasone without any response. He received further treatment with bortezomib/dexamethasone followed by autologous bone marrow transplantation. However, he experienced a further relapse 1 year later: he was treated with lenalidomide and dexamethasone, with partial and stable remission. After 13 years, he is still alive with signs of stable local disease.

### Case 2

**Fig. 1:**
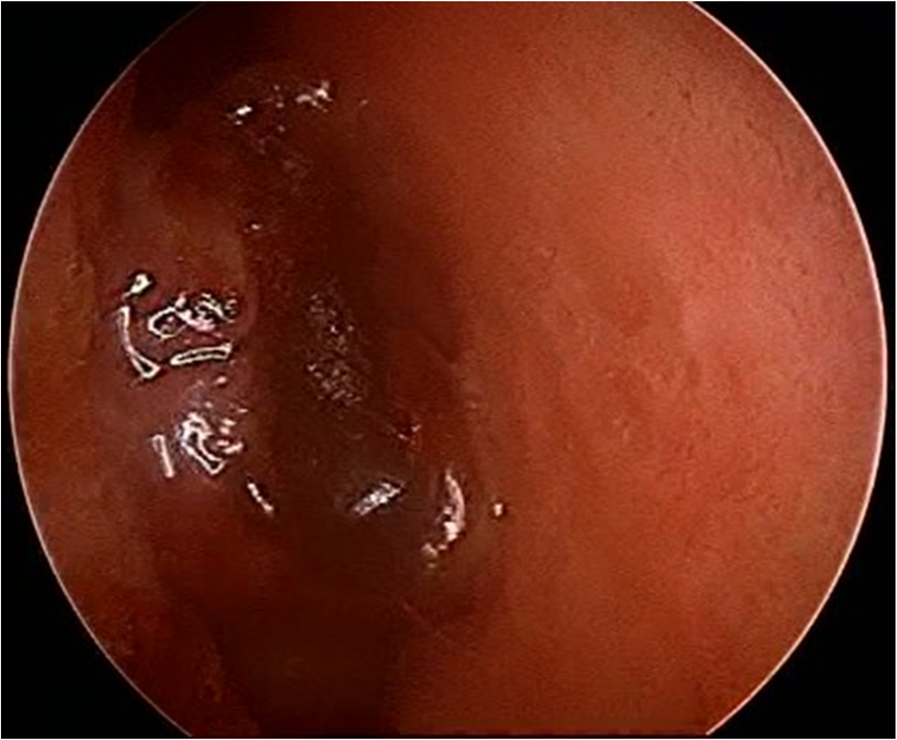
Nasal endoscopy of Case 2. Nasal endoscopy showed a bloody tumor mass occupying the right nasal cavity

A 60-year-old white man complained of right nasal respiratory obstruction and ipsilateral epistaxis and rhinorrhea. No abnormalities were evident by basal serum work-up, including serum and urine protein electrophoresis. A nasal endoscopy showed a bloody tumor mass occupying his right nasal cavity (Fig. 1); at clinical examination no palpable laterocervical lymph nodes were found.

**Fig. 2:**
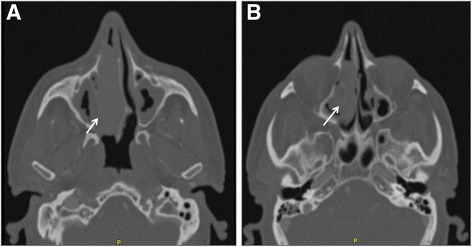
Computed tomography of Case 2. Computed tomography scan, axial view, revealed a soft tissue involving the right nasal cavity (**a**; *white arrow*), extending into the ipsilateral ethmoid sinus (**b**; *white arrow*). No signs of bone erosion were observed

A CT scan revealed a soft tissue involving his right nasal cavity, extending into the ipsilateral ethmoid sinus, not easy to excise with dimensions greater than 3.5 cm. No signs of bone erosion were observed (Fig. 2).

**Fig. 3:**
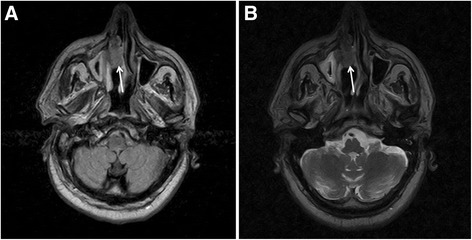
Magnetic resonance imaging with contrast of Case 2. Magnetic resonance imaging, axial view, with contrast medium showed hyperintense signal in T1 (**a**; *white arrow*) and fluid-attenuated inversion recovery sequences and hypointense signal in T2 (**b**; *white arrow*) in the right nasal fossa

MRI with contrast medium showed hyperintense signal in T1 and FLAIR sequences and hypointense signal in T2 in the right nasal fossa (Fig. 3).

A nasal biopsy performed under local anesthesia allowed diagnosis of EMP. Morphological, immunophenotypic, and immunohistochemical findings confirmed the presence of EMP with a diffuse reactivity for CD138. An iliac crest needle biopsy did not demonstrate bone marrow involvement, total body sestamibi scintigraphy showed normal tracer uptake in bones and soft tissues.

He underwent radiotherapy with a total dose of 40 Gy showing a total response without significant side effects. After 5 years he presented with recurrence localized to the left humerus bone, which was treated with radiotherapy with a total dose of 20 Gy, followed by autologous bone marrow transplantation. Three years later, a new recurrence to his right clavicle was treated with radiotherapy with total dose of 40 Gy and a further localization to his right hip appeared 1 year later and was treated with radiotherapy with total dose of 40 Gy followed by maintenance chemotherapy with thalidomide and dexamethasone with partial therapeutic response. After 12 years, is still alive with stable signs of local disease.

### Case 3

A 37-year-old white woman complained of left nasal respiratory obstruction. A monoclonal spike in serum as well as monoclonal Bence Jones protein in the urine electrophoresis were absent. Her renal functions, liver functions, and blood profile were normal. A nasal endoscopy showed a bloody tumor mass occupying her left nasal cavity; at clinical examination no palpable laterocervical lymph nodes were found.

CT and MRI revealed a mass involving her left maxillary sinus and the ipsilateral nasal cavity (size approximately 5 cm).

A nasal biopsy performed under local anesthesia confirmed the diagnosis of EMP. Immunohistochemical evaluation showed diffuse reactivity for CD138 with kappa light chain restriction. An iliac crest needle biopsy did not demonstrate bone marrow involvement.

She underwent radiotherapy for a total dose of 40 Gy, but due to progression of disease 6 months later, she underwent four cycles of chemotherapy according to the vincristine, adriamycin, and dexamethasone (VAD) scheme with only a partial response. After 2 years, because of systemic disease progression, she again received three cycles of chemotherapy according to the VAD scheme and autologous bone marrow transplantation, obtaining complete disease remission. After 12 years, she is still alive and in disease remission.

## Discussion

EMP is a rare plasma cell neoplasm which involves soft tissues, without systemic involvement. It most frequently occurs in the upper respiratory tract and oral cavity (Table 2) [2, 4, 5, 7, 10]. Since EMP can have different clinical and therapeutic features, the diagnosis of EMP and the choice of the treatment should be discussed by a multidisciplinary cancer board including an otolaryngologist, an oncohematologist, a pathologist, and a radiotherapist.

**Table 2:** Extramedullary plasmacytoma cases of nasal and paranasal sinuses reported in the English language literature

Reference	Pt	Age/Sex	Site	Symptoms	Treatment	Recurrence	Mts	Follow-up
Ashraf *et al*. 2013 [7]	3	43/M	Nasal cavity	Nasal obstruction, epistaxis	RT+surgery Surgery+RT (44 Gy) RT	No No No	No No No	1 year 1 year 3 years
Corvo *et al*. 2013 [13]	1	51/F	Nasal cavity/maxillary sinus	Nasal obstruction, epistaxis	RT (48 Gy)+surgery	No	No	6 years
Verim *et al*. 2014 [10]	1	69/F	Frontal sinus (4×3 cm)	Chronic headache	Surgery+RT (40 Gy)	No	No	18 months
D’Aguillo *et al*. 2014 [8]	175	55/ M:F of 2.3:1 M (69.4%) F (30.6%)	Nasal cavity/septum (32.5%), maxillary sinus (26.8%), nasopharynx (18.6%), ethmoid sinus (7.2%), sphenoid sinus (6.7%), paranasal sinus (6.2%), frontal sinus (2.1%)	Nasal obstruction (29.8%), epistaxis (24.2%), facial swelling (9.9%), facial pain (9.9%), painless mass (6.8%), change or loss of vision (6.2%), nasal discharge (4.3%), CN VI palsy (3.7%), proptosis/ptosis (2.5%), headache (2.5%)	RT (50.9%), surgery+RT (21.7%), surgery (14.3%), CHT (1.7%), RT+CHT (5.1%), RT+Surgery+CHT (3.4%), no therapy (2.9%)	16%	No	39–60.9 months

*CHT* chemotherapy, *CN VI palsy* sixth cranial nerve, *F* female, *M* male, *Mts* metastases, *Pt* patients, *RT* radiotherapy

In addition to biopsy and histological examination, EMP diagnostic procedures should comprise laboratory studies including serum and urine protein electrophoresis, quantitative Ig and beta-2-microglobulin determination in serum, complete nasal endoscopic examination, extensive imaging study (CT, MRI), and functional tests such as ^18^F-fluorodeoxyglucose-positron emission tomography (FDG-PET) or sestamibi scintigraphy. EMP must be distinguished from reactive plasma cell lesions and lymphoma. The diagnosis of EMP is based on monoclonal plasma cells infiltrate without B cell component. Monoclonality and/or an aberrant plasma cell phenotype should be demonstrated with useful immunophenotypic markers such as CD138, CD38, kappa/λ light chain ratio, CD19, CD56, CD27, CD117, and cyclin D1 [11].

Furthermore, bone marrow biopsy and/or needle aspiration is of utmost importance to determine the percentage of plasma cells, which should be less than 5% [1].

Regarding the natural history of the disease, Batsakis [12] defined five possible stages that EMP can present:

I. Localized disease; solitary, controlled by surgery, radiotherapy, or both; without recurrence or dissemination.
II. Disease with local recurrence controlled by additional therapy.
III. Aggressive disease, persistent or recurrent; death by uncontrollable local extension.
IV. Local disease with regional lymph node “metastasis” without evidence of distant spread.
V. Local disease, recurrent or followed by dissemination and development of another neoplasm of plasma cells and/or MM [13].

Following this classification all three of our patients are in stage V.

According to the literature, the gold standard therapy for EMP is radiotherapy [5, 8]. In fact, solitary EMPs smaller than 5 cm have an excellent local control with radiation doses of 30 to 40 Gy in 20 fractions, whereas tumors larger than 5 cm may require higher doses (40 to 50 Gy) [5]. Cervical nodes should be included if involved [5]. Overall, most studies report high local control rates of approximately 80 to 100% with moderate doses [14]. Chemotherapy is considered only in patients with tumors larger than 5 cm, high-grade tumors, refractory and/or relapsed disease, and in case of progression to MM [1, 15]. Only in small localized cases is complete surgical excision appropriate [5]. However, when clear surgical margins are obtained, the rate of local control with surgery alone is similar to that achieved with radiotherapy alone. Instead, radiotherapy followed by surgical excision is often employed to reduce the tumor volume and consequently the invasiveness of surgery [5].

Since head and neck plasma cell neoplasms can be very aggressive with a high tendency to local recurrence, it is important to adequately irradiate all cancer cells with enough doses to ensure tumor control. On the other hand, healthy tissues are very sensitive to radiation. For instance, salivary glands, larynx, and constrictor muscles can be particularly damaged by radiotherapy resulting in long-term sequelae with an incidence of acute as well as late side effects, especially regarding skin toxicity, mucositis, xerostomia, dry-eye syndrome, radiation-induced retinopathy, and neovascular glaucoma, lacrimal duct stenosis, brain necrosis, and osteoradionecrosis of the maxilla [5].

The long-term survival rate reported in the literature [8], the presence of long-term stable disease as observed in our patients, and the possibility of late recurrences suggest the need of long-term follow-up.

A recent systematic review of 175 sinonasal EMPs by D’Aguillo *et al*. [8] confirmed a higher occurrence of these neoplasms in men, with a male to female ratio of 2.3:1. They found that the mean age of diagnosis was 55 years, with a range of 5 years to 79 years. Moreover, they showed that a sinonasal EMP most often recurs as a space-occupying lesion. They found that nasal obstruction was the most common presenting symptom (29.8% of 175 cases). Other commonly cited presenting symptoms included epistaxis (24.2%), facial swelling (9.9%), facial pain (9.9%), painless mass (6.8%), and change or loss of vision (6.2%). The development of MM is a known sequelae of EMP. The rate of conversion of EMP to MM is lower than other plasma cell neoplasms, such as SPB, with rates reported to be between 11 and 33% over 10 years [8]. The highest risk of conversion is in the first 2 years after diagnosis, but conversion has been noted up to 15 years after diagnosis. Once conversion to MM is complete, less than the 10% of patients will survive 10 years. In the recent review published by D’Aguillo *et al*. [8], 16 patients (9.1%) converted to MM, with a median follow-up of 39 months (3.25 years). Eleven of these 16 patients (68.8%) died because of their disease. So it is recommended that patients receive regular follow-up after diagnosis of EMP due to the relatively high risk of conversion.

The rarity of the tumor, the lack of randomized clinical trials, and the propensity toward case reports create a debate regarding the optimal follow-up protocol for solitary EMPs of the sinonasal tract, which continues to be ambiguous.

Myeloma Guidelines by the Italian Association of Medical Oncology (AIOM) [16] suggest the first screening 45 to 60 days after radiotherapy by serum examinations, then every 3 months for the first year, subsequently every 6 months by serum, radiological, and bone marrow examinations, if necessary. Due to the high risk of conversion, D’Aguillo *et al*. [8] proposed a regular screening for MM every 6 weeks for the first 6 months after diagnosis of EMP and then periodically, but without a specific timing. For these reasons, we propose a follow-up protocol consisting of nasal endoscopy and serum examinations every 3 months, and imaging study with MRI 3 months after radiotherapy and subsequently every 6 months per year for 5 years; after 5 years, we propose serum examinations and nasal endoscopy every 6 months and MRI every year. We recommend a biopsy only in cases of clinical and instrumental suspicion of recurrence.

## Conclusions

In conclusion, due to the rarity, sites, clinical history, proximity of critical structures, as well as long-term stable disease survival, we believe that the diagnostic approach to EMP should be multidisciplinary and the treatment considered case by case, confirming, in our experience, radiotherapy as the therapy of choice. According to the literature, we observed a high (10 years) survival rate. This aspect, in addition to the possibility of recurrence and the relatively high risk for conversion to MM, prompts a thorough follow-up protocol that, in our opinion, should be designed as described.

## Abbreviations

AIOM: Italian Association of Medical Oncology
CT: Computed tomography
EMP: Extramedullary plasmacytoma
FDG-PET: ^18^F-fluorodeoxyglucose-positron emission tomography
FLAIR: Fluid-attenuated inversion recovery
MM: Multiple myeloma
MRI: Magnetic resonance imaging
SPB: Solitary plasmacytoma of bone
VAD: Vincristine, adriamycin, and dexamethasone
